# Osteofibrous dysplasia-like adamantinoma treated via intercalary segmental resection with partial cortex preservation using pedicled vascularized fibula graft: a case report

**DOI:** 10.1186/s12957-020-01983-9

**Published:** 2020-08-13

**Authors:** Yuji Yamamura, Makoto Emori, Nobuyuki Takahashi, Mitsumasa Chiba, Junya Shimizu, Yasutaka Murahashi, Shintaro Sugita, Kousuke Iba, Tadashi Hasegawa, Toshihiko Yamashita

**Affiliations:** 1 Department of Orthopedic Surgery, Sapporo Medical University School of Medicine, West 16, South 1, Chuo- ku, Sapporo, Hokkaido 060-8543 Japan; 2 Department of Diagnostic Pathology, Sapporo Medical University School of Medicine, Sapporo, Hokkaido Japan

**Keywords:** Osteofibrous dysplasia, Vascularized fibula graft, Bone tumor

## Abstract

**Background:**

Morphologically, osteofibrous dysplasia-like adamantinoma is thought to be intermediate between osteofibrous dysplasia and adamantinoma. Its treatment is not well established owing to its rarity.

**Case presentation:**

We report about of a 10-year-old girl with osteofibrous dysplasia-like adamantinoma initially diagnosed as osteofibrous dysplasia and treated via intercalary segmental resection with partial cortex preservation using a pedicled vascularized fibula graft for reconstruction. Bone union was observed 9 weeks after surgery. Twenty-two months after the definitive surgery, no recurrence was observed.

**Conclusion:**

This case illustrates the upgrade from osteofibrous dysplasia to osteofibrous dysplasia-like adamantinoma. The surgical method may aid the treatment of osteofibrous dysplasia-like adamantinoma with incomplete cortex involvement of the tumor.

## Introduction

An osteofibrous dysplasia (OFD) is a rare, benign, self-limited fibrous osseous lesion that exclusively affects the midshaft of the tibia and mostly occurs in children [1]. Pain and swelling are the most common initial symptoms. OFD usually progresses until the patient is 10-year old with stabilization at about 15 years of age and occasionally regresses spontaneously after puberty. Some lesions may be periodically aggressive with anterior tibia bowing deformity. Although most patients require close observation only, surgery is indicated when bone destruction, deformity, and local symptoms become severe.

OFD, OFD-like adamantinoma (AD), and classic AD are postulated to represent a spectrum of morphologically similar diseases. Histologically, OFD-like AD lies between OFD and AD. It was first thought to be a precursor to AD [2] but is now considered a progression of OFD [3–6]. The clinical presentation of OFD-like AD is similar to that of OFD. Typically, OFD-like AD develops during the first two decades of life, with mean age of 13.4 years [6, 7]. OFD-like AD is exclusively intracortical in the tibia with frequent synchronous involvement of the ipsilateral fibula.

The treatment of OFD-like AD is not well established owing to the scarcity of cases. It is usually based on the symptoms and the extent of the lesion, but broadly follows that for OFD with observation with plain radiographs at six monthly to annual intervals for a minimum of 10 years [6]. Although there is no evidence of progression to AD, the recommended treatment for OFD-like AD in recent years is radical segmental resection rather than curettage, as it may prevent local recurrence [5, 6]. The most popular reconstructive options after segmental excision of a bone tumor include allografts, vascularized fibula grafts (VFGs), combined allografts and VFGs, segmental endoprostheses, extracorporeal devitalized autografts, and segmental transport. A VFG is recommended to reconstruct tibial bone defects using only a VFG, but complications such as pseudarthrosis and fracture remain problematic [8].

We report a case of OFD-like AD initially diagnosed as OFD and treated via segmental resection with partial cortex preservation using a pedicled VFG (PVFG) and locking plate for reconstruction.

## Case presentation

**Fig. 1:**
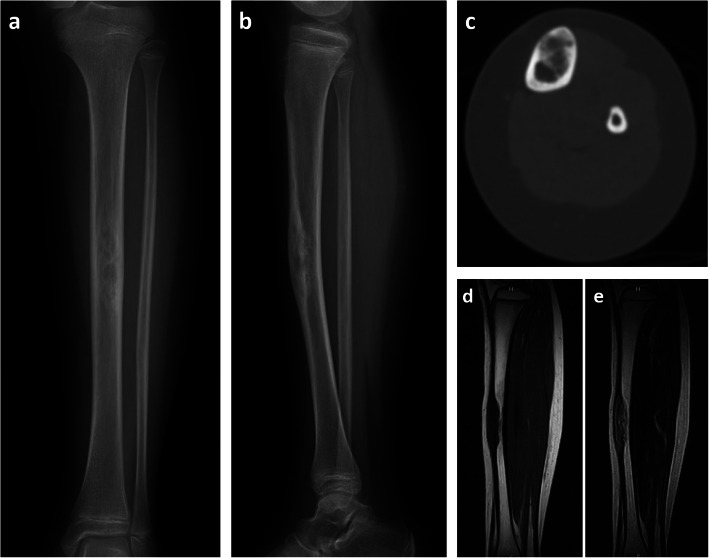
The X-ray, CT image, and MR images of the tumor. **a** Anteroposterior radiograph. **b** Lateral radiograph. **c** Axial CT image. **d** T1-weighted magnetic resonance sagittal image. **e** T2-weighted magnetic resonance sagittal image

A 10-year-old girl presented with a 3-month history of pain in her left lower leg after bruising. She had no medical conditions and was physically active. On physical examination, she had a bony lump in the middle third of the left tibia with some tenderness. An X-ray of the left leg showed a 6-cm mass with multiple osteolytic and sclerotic lesions in the thickened anterior diaphysis of the left tibia (Fig. 1a, b). No periosteal reaction was observed. Computed tomography revealed that the 6-cm mass was confined to the cortex of the tibia (Fig. 1c). On magnetic resonance imaging (MRI), it was heterogeneously hypointense and isointense on a T1-weighted sagittal image and heterogeneously hyperintense on a T2 sagittal image; it measured 63 mm in the tibia with incomplete involvement of the marrow cavity (Fig. 1d, e). The mass was highly suspected to be an OFD, and open biopsy was performed.

**Fig. 2:**
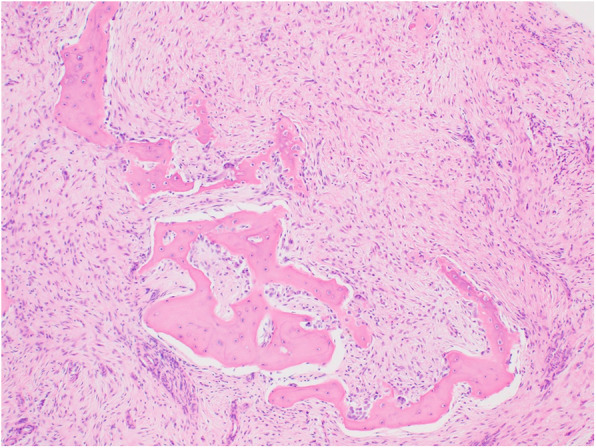
The patient was diagnosed with osteofibrous dysplasia because there was no epithelial component in the biopsy specimen

Histologic examination of the biopsy specimen revealed that immature bone trabeculae were surrounded partly by prominent osteoblastic rimming (Fig. 2). Cytokeratin (AE1/AE3)-positive epithelial cells were sparsely distributed throughout the specimen, but clusters of epithelial tissues were absent. Based on the combined results of imaging and histologic analysis, OFD was diagnosed.

**Fig. 3:**
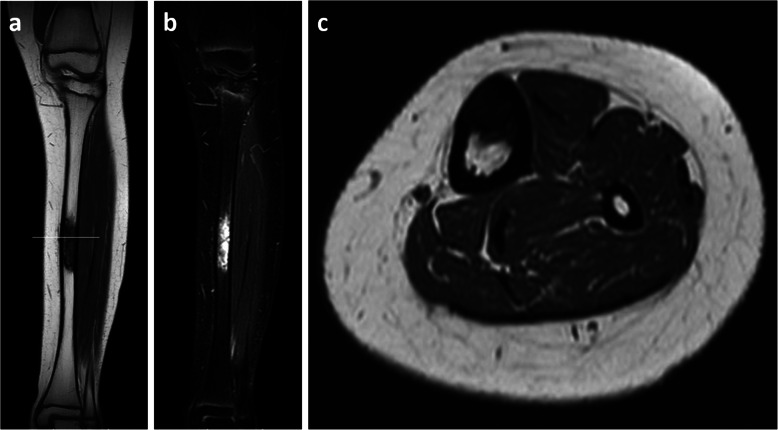
The mass was **a** heterogeneously hypointense and isointense on a T1-weighted magnetic resonance image and **b** heterogeneously hyperintense on short TI inversion recovery magnetic resonance image. No pathological fracture was confirmed

**Fig. 4:**
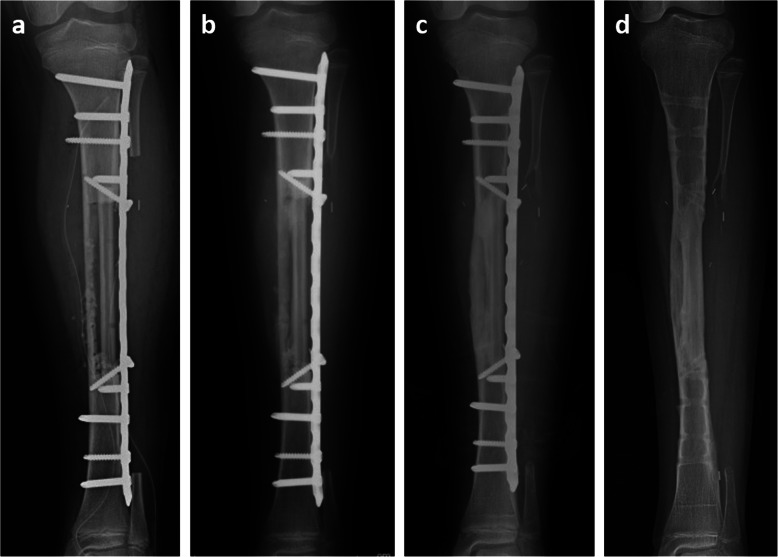
**a** A locking plate was used to mechanically align and stabilize the lower leg. A pedicled vascularized fibula graft was inserted into the medullary cavity of the tibia and locked in place with cortical screws. **b** Bone union was observed 9 weeks after surgery, and the patient began to partially bear weight. **c** Complete bone union was observed 8 months after surgery. **d** The locking plate and screws were removed 19 months after surgery

The patient was followed via observation only regardless of pain. However, she had difficulty walking owing to increasing pain 4 months after diagnosis. MRI revealed a slight increase in tumor size but no pathological fracture (Fig. 3). Because the diagnosis was OFD, her pain was first treated with painkillers but was not sufficiently controlled. We therefore decided to surgically remove the tibial tumor after thorough discussion with her parents. Nine months after OFD diagnosis, intercalary 10-cm segmental resection with 1.5-cm wide partial cortex posteromedial preservation using a PVFG and a locking plate for reconstruction was performed. Resection was preceded by osteosynthesis using a locking plate to mechanically stabilize the lower leg. A 14-cm section was removed from the patient’s fibula; after reducing its size to 12 cm, it was inlaid into medullary cavity of the tibia (Fig. 4a).

**Fig. 5:**
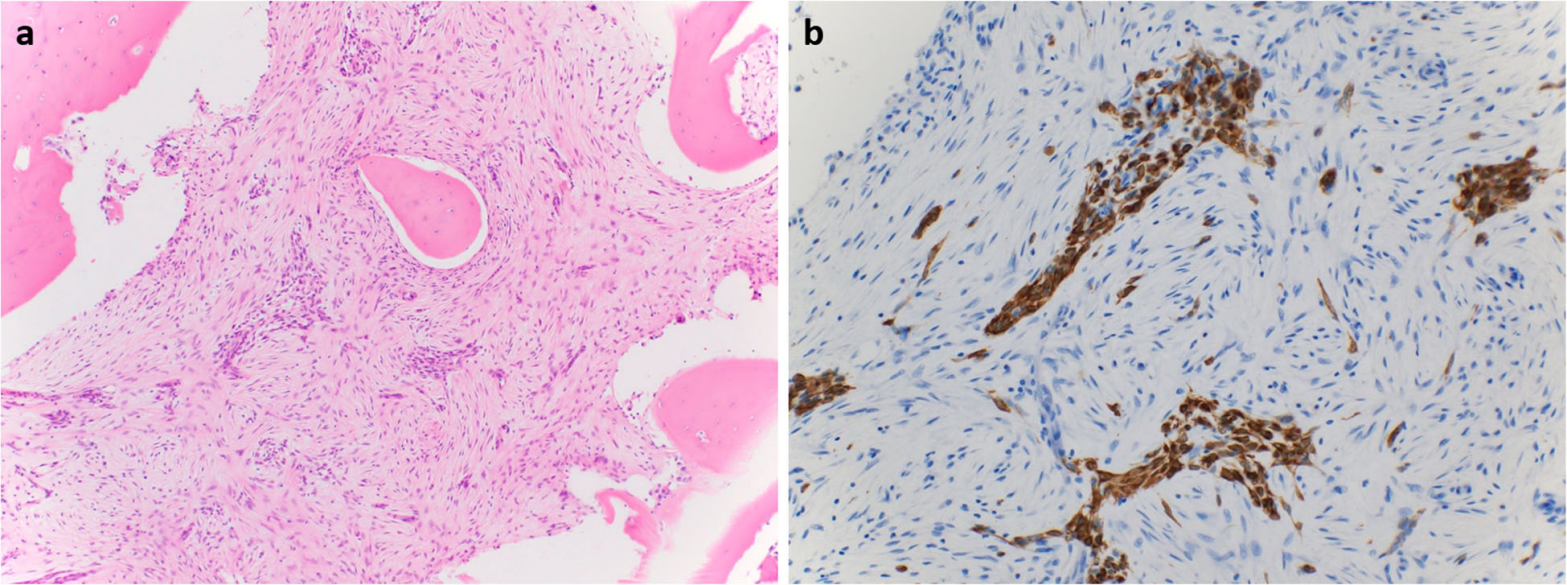
The final diagnosis was osteofibrous dysplasia-like adamantinoma because the surgical specimen contained an epithelial component (**a**) with positive AE1/AE3 immunostaining (**b**)

The tumor was composed of spindle cells with bony trabeculae rimmed by osteoblasts (Fig. 5a). Small clusters of epithelial cells were sparsely distributed throughout the tumor and were positive for AE1/AE3 immunostaining (Fig. 5b). Therefore, the diagnosis was upgraded to OFD-like AD. Bone union was observed 9 weeks after surgery (Fig. 4b), at which time the patient was allowed to partially bear weight. Full weight-bearing was allowed 12 weeks postoperatively. No complications were observed. Eight months after surgery (Fig. 4c), the patient walks without a cane. Nineteen months after surgery, the plate was removed (Fig. 4d). Twenty-two months after the definitive surgery, no recurrence was observed.

## Discussion

This case is of interest to clinicians for two reasons. First, it shows progression of OFD to OFD-like AD. Second, it describes the successful treatment of OFD-like AD via segmental resection with partial cortex preservation using a PVFG and a locking plate for reconstruction. This surgical method may achieve earlier bone union than do other methods and be feasible for cases of OFD and OFD-like AD in which the cortex can be partially preserved.

In our case, the diagnosis was changed from OFD to OFD-like AD after examination of a surgical specimen. Progressive disease requiring surgery in patients with OFD-like AD initially treated for OFD has been reported [6, 7]. Therefore, open biopsy is preferred to needle biopsy as it may better detect the small sparse clusters of epithelial cells that differentiate OFD-like AD from OFD. We performed open biopsy to prevent sampling errors, and the initial diagnosis was OFD [7]. The upgrade from OFD to OFD-like AD may represent a sampling error, but it is likely that disease progression had occurred. Patients with OFD may present with localized pain (25–60%) [9, 10]. About 80% of OFD-like AD cases are accompanied by pain [6], and it is possible that tumors diagnosed as OFDs are actually OFD-like ADs in cases with severe pain.

Although our patient was initially diagnosed with OFD, we performed segmental resection because we could not control her pain. Several articles in recent years recommend radical segmental resection of OFDs or OFD-like ADs to prevent local recurrence [6, 7, 11]. The most popular reconstructive options after segmental resection of a bone tumor include intercalary allografts, VFGs, combined allografts and VFGs, endoprostheses, extracorporeal devitalized (i.e., irradiated, frozen, or pasteurized) autografts, and segmental transport based on the principles of distraction osteogenesis.

Intercalary allografts, the most widely accepted option, have a high incidence of nonunion, fracture, and infection. The use of a single devitalized autograft is also associated with nonunion. Therefore, for intercalary reconstruction, we prefer VFGs, which had good functional and oncological outcomes in the previous work of Emori et al. [8]. In VGF-mediated reconstruction, bone union in the leg usually takes 5–7 months, but complications such as pseudarthrosis and fracture may arise. Hence, a strong fixation device such as a locking plate should be used to prevent these complications [8, 12]. In the present case, segmental resection with partial cortex preservation using a PVFG and a locking plate expedited bone union (within 9 weeks after surgery) without complications, presumably because the partially preserved cortex better stabilized the affected limb.

## Conclusions

We report a case of OFD-like AD that progressed from OFD and was treated via segmental resection with partial cortex preservation using a PVFG and a locking plate for reconstruction. This surgical method may provide early bone union and be feasible for cases of OFD and OFD-like AD in which the cortex can be partially preserved because of incomplete cortex involvement.

## Data Availability

All data obtained is available within the manuscript.

## Abbreviations

OFD: Osteofibrous dysplasia
AD: Adamantinoma
VFG: Vascularized fibula graft
